# The Role of Impulse Oscillometry in Detection of Preserved Ratio Impaired Spirometry (PRISm)

**DOI:** 10.3390/arm93010002

**Published:** 2025-01-27

**Authors:** Chalerm Liwsrisakun, Warawut Chaiwong, Athavudh Deesomchok, Pilaiporn Duangjit, Chaicharn Pothirat

**Affiliations:** Division of Pulmonary, Critical Care, and Allergy, Department of Internal Medicine, Faculty of Medicine, Chiang Mai University, Chiang Mai 50200, Thailand; chalerm.liw@cmu.ac.th (C.L.); athavudh.d@cmu.ac.th (A.D.); pilaiporn.th@cmu.ac.th (P.D.); chaicharn.p@cmu.ac.th (C.P.)

**Keywords:** impulse oscillometry, spirometry, small airway disease, resistance, reactance

## Abstract

**Highlights:**

**What are the main findings?**

- The R5-R20 variable in impulse oscillometry showed an acceptable performance for detecting PRISm.

**What are the implications of the main findings?**

- PRISm may be detected by impulse oscillometry (IOS) especially in subjects who cannot perform spirometry.

**Abstract:**

Background: Information is limited regarding the role of impulse oscillometry (IOS) for the detection of preserved ratio impaired spirometry (PRISm). Therefore, we aimed to study the diagnostic ability of IOS in differentiating between PRISm and healthy subjects. Methods: This retrospective data collection was done at the Lung Health Center, Faculty of Medicine, Chiang Mai University, Thailand between July 2019 and April 2022. The potential diagnostic possibilities of difference in resistance at 5 Hz (R5) and resistance at 20 Hz (R20) (R5-R20) for PRISm detection were analyzed. Results: The prevalence of PRISm was higher when using the fixed ratio (FR) criteria (FEV_1_/FVC ≥0.7 with FEV_1_ < 80% of predicted value) compared to the lower limit of normal (LLN) criteria (FEV_1_/FVC ≥ LLN and FEV_1_ < LLN) (10.0% vs. 4.2%). The %prediction for R5-R20 provided an acceptable area under the curve (AUC) for PRISm, defined by the LLN and the FR criteria (AuROC = 0.75 (95%CI; 0.64, 0.85) and 0.72 (95%CI; 0.63, 0.81), respectively). The cut-off value of %predicted R5-R20 ≥120% resulted in the highest sensitivity and specificity for detecting PRISm. Conclusions: The %predicted of R5-R20 ≥ 120% showed an acceptable performance for PRISm detection and PRISm may be detected by IOS.

## 1. Introduction

Preserved ratio impaired spirometry (PRISm) is defined by a decline in forced expiratory volume in the first second (FEV_1_) without evidence of spirometry-defined airflow obstruction [1]. PRISm is associated with morbidity and mortality caused by respiratory and cardiovascular diseases [2,3,4,5,6,7,8]. The estimated global prevalence varies from 4.7% to 22.3% due to differences in the definition used [9,10,11,12,13]. These definitions include FEV_1_/forced vital capacity (FVC) above the statistically defined fifth percentile of normal (lower limit of normal; LLN) but with FEV_1_ < LLN, or the fixed ratio (FR) criteria (FEV_1_/FVC ≥ 0.7) with FEV_1_ < 80% of predicted value [9].

Impulse oscillometry (IOS) is a non-invasive tool designed for evaluation of small airway dysfunction (SAD) [14]. IOS has become a more practical method, particularly for the elderly with cognitive impairment, who may not accurately perform forced exhalation like spirometry [15]. Unacceptable spirometry results are common in clinical practice, ranging from 8.9–19.8% [16,17]. Furthermore, IOS can be used as an effective screening method for chronic obstructive pulmonary disease (COPD). Chaiwong et al. found that IOS was a valuable tool for the diagnosis of COPD with sensitivity and specificity close to 80% [18]. Additionally, previous studies have found that PRISm is associated with SAD when compared to individuals with normal spirometry [9,19]. Ding et al. reported that IOS parameters were higher in COPD and PRISm compared to healthy controls [19]. However, these studies used absolute values of IOS parameters for comparison. IOS variables are influenced by some factors including age, sex, height, and body weight [20]. Therefore, in this study the aim was to compare IOS parameters both as absolute values and as a percentage of predicted values in subjects with PRISm, COPD, and healthy subjects. We also focused on the diagnostic contribution of IOS in differentiating between PRISm and healthy subjects.

## 2. Materials and Methods

### 2.1. Study Procedures

This retrospective study, including data from subjects with COPD, asthma, post-coronavirus disease 2019 (COVID-19) and a comparable control healthy population was done at the Lung Health Center, Department of Internal Medicine, Faculty of Medicine, Chiang Mai University, Chiang Mai, Thailand [18,20,21,22]. We collected spirometry and IOS results measured from July 2019 to April 2022. Only spirometry results meeting the American Thoracic Society (ATS)/European Respiratory Society (ERS) standards [23] and IOS results meeting ERS recommendations [14] were included in the analysis. We also recorded demographic data including age, sex, height, body weight, body mass index (BMI), underlying diseases and smoking status. The Research Ethics Committee of the Faculty of Medicine, Chiang Mai University (Institutional Review Board (IRB) approved this study with the approval number: MED-2567-0508, approval date: 3 September 2024). Due to the retrospective nature of this study, written informed consent was waived.

### 2.2. Definition

PRISm is characterized by two criteria: (1) the LLN criteria (FEV_1_/FVC ≥ LLN and FEV_1_ < LLN) and (2) the FR criteria (FEV_1_/FVC ≥ 0.7 and FEV_1_ < 80% of predicted value). COPD in this study was classified by the diagnostic criteria from ATS/ERS using a post- bronchodilator (BD) FEV_1_/FVC ratio below LLN [23] and Global Initiative for Chronic Obstructive Lung Disease (GOLD) criteria using a FR of post-BD FEV_1_/FVC ratio below 0.7 [24]. Healthy subjects were a population with no chronic respiratory symptoms, no previous diagnosis of any chronic respiratory diseases by physicians, being lifelong non-smokers and no evidence of abnormal spirometry results. They were used as a control group.

### 2.3. Spirometry and Impulse Oscillometry (IOS)

All pre-BD IOS and spirometry results were obtained using the Vmax 22 spirometer (CareFusion, Hoechberg, Germany). The recorded parameters included FVC, FEV_1_, FEV_1_/FVC ratio and the average expired flow over the middle half (25–75%) of the FVC maneuver (FEF25-75%). The Global Lung Initiative (GLI) 2012 reference equations for the Southeast Asian sub-group were used to calculate the predicted values for all spirometry parameters [25]. We reported the absolute values, %predicted values and z-scores for FVC, FEV_1_, the FEV_1_/FVC ratio and FEF25-75%.

IOS parameters, including resistance at 5 Hz (R5), resistance at 20 Hz (R20), the difference in resistance (R5-R20), resonant frequency (Fres), reactance at 5 Hz (X5) and the area under the reactance curve between 5 Hz and the resonant frequency (AX) were also collected. Both absolute values and %predicted values, calculated by using Thai predictive values [20], were reported.

### 2.4. Statistical Analysis

Results for continuous data were shown as mean ± standard deviation (SD) or medians with interquartile ranges (IQR), depending on distribution. Results for categorical data were shown as frequencies and percentages. One-way analysis of variance (ANOVA) with the Bonferroni adjustment method and the Kruskal–Wallis test were used to analyze differences across the three groups for parametric and non-parametric data, respectively. In case of non-parametric data, the Mann–Whitney U test was used to compare differences between the two groups. Chi-square and Fisher’s exact tests were used to compare categorical data across the three groups and between two groups, respectively. Statistical significance was set at a *p*-value < 0.05. For multiple comparisons, the adjusted level of significance was estimated by dividing the significance level by the number of comparisons among the three groups. Thus, the *p*-value for multiple comparisons was set at 0.017 (0.05/3).

Agreement on the categorization of PRISm, normal spirometry and obstructive airway (OA) between the LLN and the FR criteria was analyzed using kappa (κ). Kappa values of 0.61–0.80 and 0.81–1.00 were interpreted as good and very good agreement, respectively [26]. Additionally, percentages of agreement for these categorizations were also calculated.

The receiver operating characteristic (ROC) curve was plotted to assess PRISm detection using the area under the curve (AUC) and 95% confidence interval (CI). Sensitivity, specificity, positive likelihood ratio (+LR), negative likelihood ratio (-LR) and diagnostic odds ratio from the %predicted of R5-R20 were calculated to identify the optimal cut-off point for PRISm detection. All statistical analyses were performed using STATA version 16 (StataCorp, College Station, TX, USA).

## 3. Results

**Figure 1:**
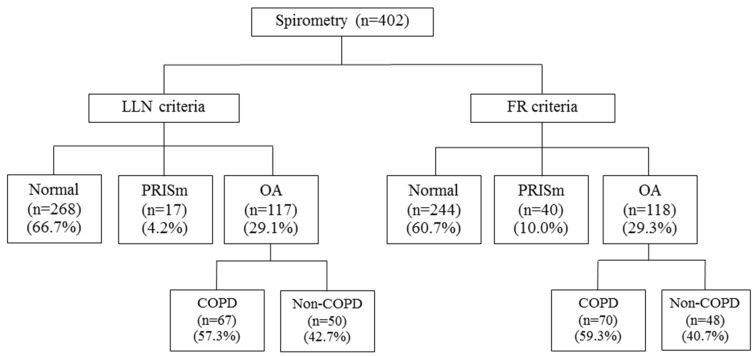
Study flow chart. **Abbreviations:** LLN, lower limit of normal, FR, fixed ratio; OA, obstructive airway; PRISm, preserved ratio impaired spirometry; COPD, chronic obstructive pulmonary disease.

Spirometry results from four hundred and two subjects were included to enable an agreement analysis on the categorization of PRISm, normal spirometry and obstructive airway (OA) according to the FR criteria and the LLN criteria. The prevalence of PRISm was higher when using the FR criteria compared to the LLN criteria (10.0% vs. 4.2%) (Figure 1). Good agreement (kappa value 0.79 (95% CI; 0.73, 0.85)) and a high percentage of agreement (89.3%) were observed for the categorization of PRISm, normal spirometry and OA between the LLN and the FR criteria.

Baseline characteristics of PRISm and COPD subjects defined using the LLN criteria and the FR criteria as well as characteristics of healthy subjects are shown in Table 1 and Table 2, respectively. There were significant differences in age, proportion of male sex, body weight, BMI, smoking status and underlying diseases, including cardiovascular and metabolic diseases, across the three groups. BMI was significantly higher in the PRISm group. Cardiovascular and metabolic diseases were more common in the PRISm and COPD groups compared to the healthy controls.

Spirometry and IOS results from the PRISm and COPD groups defined by using the LLN criteria, and the FR criteria when compared to healthy subjects are shown in Table 3 and Table 4, respectively. A total of 164 and 190 subjects were included in the analysis as defined by the LLN and the FR criteria, respectively. When the LLN criteria were used, the number of subjects in the PRISm and COPD were 17 and 67, respectively. When using the FR criteria, the number of participants in each group were 40 and 70, respectively. The LLN criteria resulted in all spirometric parameters in PRISm being significantly higher than COPD with the exception of the absolute value and %predicted value of FEV_1_ and FVC (Table 3). All spirometric parameters in PRISm were also significantly higher compared to COPD except for the absolute value, z-score, and %predicted of and FVC when using the FR criteria (Table 4). All spirometric parameters in the PRISm group were significantly lower than the healthy subjects with the exception of the FEV_1_/FVC (%) and the z-score of FEV_1_/FVC for both criteria (Table 3 and Table 4).

A significant increase in all parameters of IOS except for the absolute value of R5 and R20 were observed in the COPD group compared to the PRISm group. Absolute value and %predicted of R5-R20 in the PRISm group were significantly higher than the healthy subjects for both criteria. In the FR criteria, a significant increase in the absolute value and % predicted of Fres were found in the PRISm group compared to the healthy subjects. A significant decrease in X5 was seen in the COPD group compared to both the PRISm and control group in both criteria. More data are shown in Table 3 and Table 4.

**Figure 2:**
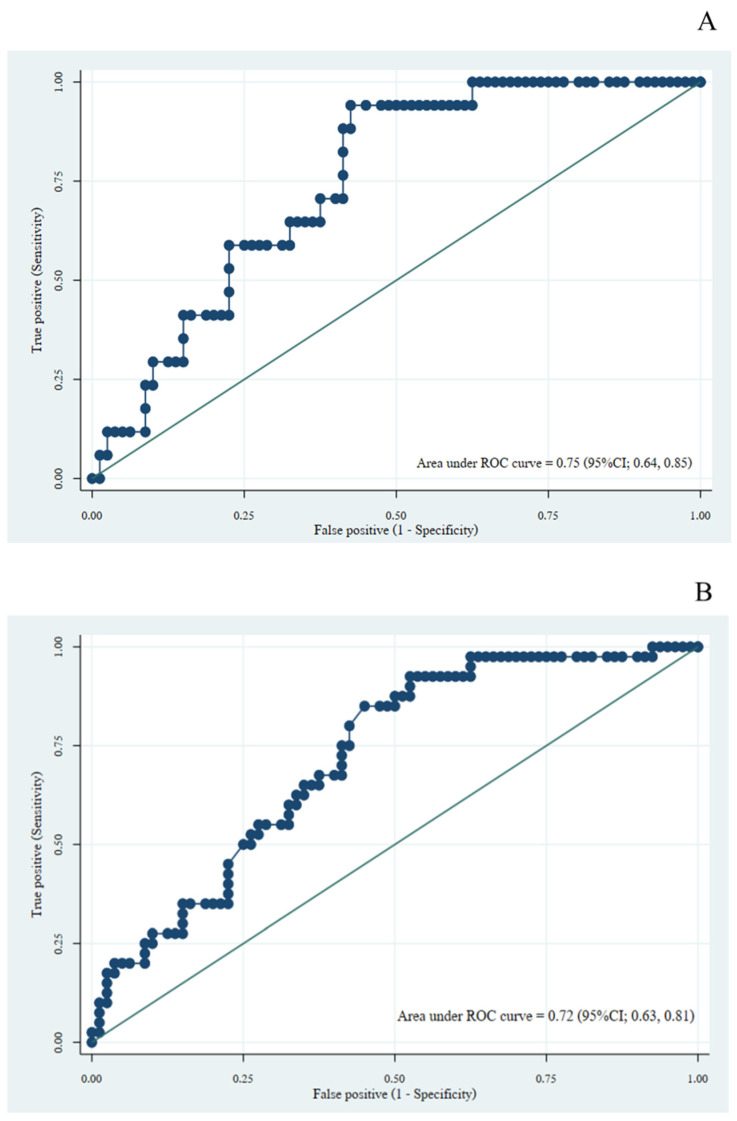
Receiver Operating Characteristic (ROC) Curves of R5-R20 for Detection of PRISm. **Note:** (**A**) lower limit of normal criteria; (**B**) fixed ratio criteria.

The areas under the receiver operating characteristic (AuROC) curve were compared between PRISm and healthy subjects. Only %predicted of R5-R20 demonstrated an acceptable accuracy relative to the detection of PRISm using both LLN and FR criteria with an AuROC of 0.75 (95%CI; 0.64, 0.85) and 0.72 (95%CI; 0.63, 0.81), respectively. More data are shown in Figure 2 and Table 5.

The cut-off value of %predicted R5-R20 ≥ 120 exhibited the highest sensitivity and specificity for detecting PRISm for both criteria, with a sensitivity of 70.6% and a specificity of 60.0% for the LLN criteria and a sensitivity of 67.5% and a specificity of 60.0% for the FR criteria. More data are shown in Table 6.

## 4. Discussion

Our study observed a higher airway resistance measured by %predicted of R5-R20 in PRISm individuals compared to healthy subjects. We noted that the %predicted of R5-R20 demonstrated an acceptable diagnostic ability for detection of PRISm. It is evident that the cut-off value of %predicted R5-R20 ≥120 may be used for detecting PRISm.

Our study found that the prevalence of PRISm was higher when using the FR criteria compared to the LLN criteria (10.0% vs. 4.2%). However, a high agreement was observed for the categorization of PRISm, normal spirometry and OA between the LLN and the FR criteria. As all lung function parameters decline with age, as expected, the predicted value and LLN for FEV_1_/FVC were higher in younger adults compared to older individuals. Therefore, using the FR value of FEV_1_/FVC is often overestimated in the elderly and underestimated in the young [27,28]. The relatively younger age in our subjects resulted in a higher prevalence of PRISm by using FR criteria.

BMI was significantly higher in the PRISm group, which was supported by a previous study which reported that BMI was higher in PRISm compared to subjects with OA and healthy individuals [3]. That study also found that PRISm was associated with both overweight and obesity [3]. Respiratory dysfunction in obesity is caused by multiple factors, including fat accumulation on the chest, decreased chest wall compliance, increased respiratory workload due to fat deposition between muscles, and non-smoking-related peripheral airway obstruction [10]. Cardiovascular and metabolic diseases were significantly more common in the PRISm and COPD groups compared to healthy subjects, observations consistent with findings in previous studies regarding the association between PRISm and diabetes mellitus (DM) and also cardiovascular comorbidities [3,9]. Pulmonary fibrosis, lung parenchymal damage, and structural alterations in DM are caused by microvascular changes, chronic inflammation, autonomic neuropathy, and loss of pulmonary elasticity due to collagen glycation [10,29]. In cardiovascular diseases, in particular hypertension, lung compliance and function are impacted by left ventricular function, pulmonary interstitial edema, and pulmonary arterial pressures [10]. These changes might explain the spirometric findings of PRISm in both groups of diseases.

We found that most of the IOS parameters including R5-R20, Fres, and AX were significantly higher in PRISm (defined by both the LLN and the FR criteria) and COPD groups compared to healthy subjects. Significantly higher %predicted and absolute values of R5-R20, Fres and AX were also observed in the COPD group when compared to the PRISm group. Previous studies also found that the absolute values of all IOS parameters were significantly higher in PRISm and COPD cases compared to healthy individuals [9,19]. These might suggest an initial impairment of small airways in PRISm before progressing to COPD [19].

To the best of our knowledge, this study is the first to demonstrate the diagnostic contribution of IOS parameters for detecting PRISm using the %predicted values. We found that the %predicted of R5-R20 provided an acceptable AUC for detection of PRISm, as defined by the LLN and the FR criteria. Additionally, the cut-off value of %predicted R5-R20 ≥120 exhibited the highest sensitivity, specificity and diagnostic odds ratio for the detection of PRISm. A previous study found that the AUC for PRISm detection using SAD indicators of spirometry like FEF25-75% was relatively low (AUC < 0.7) [30]. Our study demonstrated that the R5-R20 of the IOS parameters had a higher AUROC at an acceptable level for detection of PRISm defined by both criteria. Therefore, it is reasonable to conclude that the use of IOS may provide adequate information for the detection of PRISm.

The strength of our study lies in its role as the first to identify the utility of IOS for the detection of PRISm using both LLN and FR criteria. However, the study has some limitations. First, it is a single-center study, the cut-off value of %predicted R5-R20 for detecting PRISm may vary in different settings. Second, Thai IOS reference values were used for % predicted calculation; thus, the generalizability of this cut-point may be limited in other populations. Third, only pre-BD IOS was measured in our study. In future studies both pre- and post-BD need to be included. Lastly, some factors that could affect the results, such as radiographic findings, air pollution and occupational exposures were not considered. Thus, again, these variations need to be factored in in future studies.

## 5. Conclusions

The %predicted R5-R20 ≥120 from IOS study showed an acceptable performance for detection of PRISm defined by both LLN and FR criteria. PRISm may be detected by IOS especially in subjects who cannot perform a forced maneuver.

## Figures and Tables

**Table 1:** Baseline Characteristics of PRISm and COPD defined by using the LLN criteria and Healthy Subjects (*n* = 164).

Clinical Characteristics	PRISm LLN Criteria (*n* = 17)	COPD LLN Criteria (*n* = 67)	Healthy Subjects (*n* = 80)	*p*-Value
Age (year)	47.7 ± 19.8	69.1 ± 8.5 *	52.2 ± 15.8 ^#^	<0.001
Male sex, *n* (%)	7 (41.2)	59 (88.1) *	41 (51.2) ^#^	<0.001
Height (cm)	158.5 ± 9.0	158.9 ± 7.0	158.9 ± 8.6	0.970
Body weight (kg)	73.4 ± 22.1	54.4 ± 12.4 *	61.5 ± 12.5 *	<0.001
Body mass index (BMI, kg/m^2^)	28.9 ± 7.1	22.6 ± 4.4 *	24.2 ± 3.6 *^,#^	<0.001
Smoking status				<0.001
Non-smoker	13 (76.5)	0 (0.0) *	80 (100.0) ^#^	
Ex-smoker	4 (23.5)	65 (97.0) *	0 (0.0)	
Current-smoker	0 (0.0)	2 (3.0)	0 (0.0)	
Smoking pack-year (median, IQR)	0.0 (0.0, 0.0)	25.0 (16.4, 42.0) *	0.0 (0.0, 0.0)^#^	<0.001
Comorbidities, *n* (%)				
Cardiovascular disease	11 (64.7)	40 (59.7)	11 (13.8) *^,#^	<0.001
Metabolic disease	4 (23.5)	13 (19.4)	1 (1.2) *^,#^	<0.001
Neuromuscular disease	1 (5.9)	6 (9.0)	0 (0.0) ^#^	0.026

**Note:** Data are mean ± standard deviation (SD) unless otherwise stated; *p*-value from analysis of variance (ANOVA) or Chi-square; *, *p* < 0.017 compared with PRISm; ^#^, *p* < 0.017 compared with COPD; *p*-value of difference between group was significant with adjusted level of significance; (0.05/3 = 0.017). **Abbreviations:** PRISm, preserved ratio impaired spirometry; COPD, chronic obstructive pulmonary disease; IQR, interquartile range; LLN, lower limit of normal.

**Table 2:** Baseline Characteristics of PRISm and COPD defined by using the FR criteria compared to Healthy Subjects (*n* = 190).

Clinical Characteristics	PRISm FR Criteria (*n* = 40)	COPD FR Criteria (*n* = 70)	Healthy Subjects (*n* = 80)	*p*-Value
Age (year)	54.8 ± 16.3	69.3 ± 8.5 *	52.2 ± 15.8 ^#^	<0.001
Male sex, *n* (%)	18 (45.0)	62 (88.6) *	41 (51.2) ^#^	<0.001
Height (cm)	158.0 ± 9.4	159.1 ± 7.0	158.9 ± 8.6	0.774
Body weight (kg)	66.1 ± 14.1	57.5 ± 12.8 *	61.5 ± 12.5	0.004
Body mass index (BMI, kg/m^2^)	26.4 ± 4.8	22.6 ± 4.4 *	24.2 ± 3.6 *^,#^	<0.001
Smoking status				<0.001
Non-smoker	33 (82.5)	0 (0.0) *	80 (100.0) *^,#^	
Ex-smoker	7 (17.5)	68 (97.1) *	0 (0.0)	
Current-smoker	0 (0.0)	2 (2.9)	0 (0.0)	
Smoking pack-year (median, IQR)	0.0 (0.0, 0.0)	25.4 (16.4, 42.5) *	0.0 (0.0, 0.0) ^#^	<0.001
Comorbidities, *n* (%)				
Cardiovascular disease	13 (32.5)	42 (60.0) *	11 (13.8) *^,#^	<0.001
Metabolic disease	7 (17.5)	14 (20.0)	1 (1.2) *^,#^	0.001
Neuromuscular disease	2 (5.0)	6 (8.6)	0 (0.0) ^#^	0.032

**Note:** Data are mean ± standard deviation (SD) unless otherwise stated; *p*-value from analysis of variance (ANOVA) or Chi-square; *, *p* < 0.017 compared with PRISm; ^#^, *p* < 0.017 compared with COPD; *p*-value of difference between group was significant with adjusted level of significance; (0.05/3 = 0.017). **Abbreviations:** PRISm, preserved ratio impaired spirometry; COPD, chronic obstructive pulmonary disease; IQR, interquartile range; FR, fixed ratio.

**Table 3:** Spirometric and IOS data of PRISm and COPD defined by using the LLN criteria compared to Healthy Subjects (*n* = 164).

Spirometry Data (Pre-Bronchodilator)	PRISm LLN Criteria (*n* = 17)	COPD LLN Criteria (*n* = 67)	Healthy Subjects (*n* = 80)	*p*-Value
FVC (Liter)	2.18 ± 0.57	2.35 ± 0.64	3.09 ± 0.82 *^,#^	<0.001
%predicted FVC	71.5 ± 7.4	82.2 ± 18.8	100.1 ± 15.1 *^,#^	<0.001
z-score of FVC	−2.03 ± 0.51	−1.09 ± 1.14 *	0.09 ± 0.09 *^,#^	<0.001
FEV_1_ (Liter)	1.81 ± 0.58	1.36 ± 0.46	2.53 ± 0.71 *^,#^	<0.001
%predicted FEV_1_	69.8 ± 7.5	59.8 ± 18.5	99.8 ± 12.1 *^,#^	<0.001
z-score of FEV_1_	−2.05 ± 0.37	−2.27 ± 1.01	−0.02 ± 0.81 *^,#^	<0.001
FEV_1_/FVC (%)	82.2 ± 7.1	57.4 ± 9.0 *	81.6 ± 5.5 ^#^	<0.001
z-score of FEV_1_/FVC	−0.28 ± 0.76	−2.88 ± 1.09 *	−0.17 ± 0.75 ^#^	<0.001
FEF25-75% (Liter/sec)	2.19 ± 1.38	0.64 ± 0.28 *	2.78 ± 1.12 *^,#^	<0.001
%predicted FEF25-75%	74.6 ± 22.4	31.3 ± 13.7 *	102.3 ± 24.1 *^,#^	<0.001
z-score of FEF25-75%	−0.99 ± 0.87	−2.50 ± 0.79 *	0.04 ± 0.84 *^,#^	<0.001
**IOS parameters (Pre-bronchodilator)**				
R5 (cmH_2_O/L/s)	4.48 ± 1.79	4.78 ± 1.60	3.77 ± 1.31 ^#^	<0.001
% predicted R5	99.7 ± 33.9	158.3 ± 59.2 *	99.8 ± 25.3 ^#^	<0.001
R20 (cmH_2_O/L/s)	3.22 ± 1.24	3.11 ± 0.98	3.13 ± 0.95	0.923
% predicted R20	88.3 ± 28.8	121.3 ± 34.8 *	98.6 ± 20.5 ^#^	<0.001
R5-R20 (cmH_2_O/L/s) (median, IQR)	0.96 (0.76, 1.72)	1.57 (0.86, 2.12) *	0.58 (0.25, 0.88) *^,#^	<0.001
% predicted R5-R20 (median, IQR)	175.9 (108.9, 271.3)	282.1 (171.8, 412.8) *	98.5 (51.5, 161.4) *^,#^	<0.001
X5 (cmH_2_O/L/s) (median, IQR)	−0.93 (−2.03, −0.65)	−1.96 (−2.68, −1.34) *	−0.98 (−1.36, −0.54) ^#^	<0.001
% predicted X5 (median, IQR)	99.6 (53.5, 204.1)	189.9 (141.1, 279.8) *	95.5 (59.9, 138.3) ^#^	<0.001
Fres (Hz)	16.2 ± 5.8	22.9 ± 5.5 *	12.8 ± 4.2 ^#^	<0.001
% predicted Fres	117.0 ± 33.4	169.3 ± 47.7 *	100.8 ± 28.4 ^#^	<0.001
AX (cmH_2_O/L) (median, IQR)	4.76 (3.35, 12.11)	17.10 (8.99, 26.12) *	3.96 (2.13, 5.99) ^#^	<0.001
% predicted AX (median, IQR)	167.1 (124.9, 244.3)	459.7 (234.9, 779.0) *	116.9 (61.8, 186.5) ^#^	<0.001

**Note:** Data are mean ± standard deviation (SD) unless otherwise stated; *p*-value from analysis of variance (ANOVA) or Chi-square; *, *p* < 0.017 compared with PRISm; ^#^, *p* < 0.017 compared with COPD; *p*-value of difference between group was significant with adjusted level of significance; (0.05/3 = 0.017). **Abbreviations:** IOS, impulse oscillometry; PRISm, preserved ratio impaired spirometry; COPD, chronic obstructive pulmonary disease; FVC, forced vital capacity; FEV_1_, forced expiratory volume in the first second; FEF25-75%, forced expiratory flow at 25–75% of FVC; LLN, lower limit of normal; R5, resistance at 5 Hz; R20, resistance at 20 Hz; R5-R20, heterogeneity of resistance between R5 and R20; Fres, resonant frequency; X5, reactance at 5 Hz; AX, the area under reactance curve between 5 Hz and resonant frequency.

**Table 4:** Impulse Oscillometry (IOS) Data of PRISm and COPD defined by using the FR criteria compared to Healthy Subjects (*n* = 190).

Spirometry Data (Pre-Bronchodilator)	PRISm FR Criteria (*n* = 40)	COPD FR Criteria (*n* = 70)	Healthy Subjects (*n* = 80)	*p*-Value
FVC (Liter)	2.27 ± 0.63	2.38 ± 0.64	3.09 ± 0.82 *^,#^	<0.001
%predicted FVC	77.5 ± 8.6	82.8 ± 19.1	100.1 ± 15.1 *^,#^	<0.001
z-score of FVC	−1.54 ± 0.66	−1.06 ± 1.16	0.09 ± 0.09 *^,#^	<0.001
FEV_1_ (Liter)	1.79 ± 0.53	1.39 ± 0.46 *	2.53 ± 0.71 *^,#^	<0.001
%predicted FEV_1_	73.5 ± 6.6	60.8 ± 18.9 *	99.8 ± 12.1 *^,#^	<0.001
z-score of FEV_1_	−1.70 ± 0.52	−2.22 ± 1.04 *	−0.02 ± 0.81 *^,#^	<0.001
FEV_1_/FVC (%)	78.8 ± 6.3	57.8 ± 9.1 *	81.6 ± 5.5 ^#^	<0.001
z-score of FEV_1_/FVC	−0.60 ± 0.79	−2.82 ± 1.10 *	−0.17 ± 0.75 ^#^	<0.001
FEF25-75% (Liter/sec)	1.79 ± 0.98	0.66 ± 0.29 *	2.78 ± 1.12 *^,#^	<0.001
%predicted FEF25-75%	68.9 ± 19.6	32.4 ± 14.6 *	102.3 ± 24.1 *^,#^	<0.001
z-score of FEF25-75%	−1.14 ± 0.71	−2.45 ± 0.82 *	0.04 ± 0.84 *^,#^	<0.001
**IOS parameters (Pre-bronchodilator)**				
R5 (cmH_2_O/L/s)	4.63 ± 1.83	4.67 ± 1.65	3.77 ± 1.31 ^#^	0.001
% predicted R5	113.1 ± 49.5	155.6 ± 59.7 *	99.8 ± 25.3 ^#^	<0.001
R20 (cmH_2_O/L/s)	3.38 ± 1.23	3.06 ± 0.99	3.13 ± 0.95	0.278
% predicted R20	101.5 ± 47.8	120.1 ± 35.2 *	98.6 ± 20.5 ^#^	<0.001
R5-R20 (cmH_2_O/L/s) (median, IQR)	0.89 (0.61, 1.69)	1.54 (0.84, 2.11) *	0.58 (0.25, 0.88) *^,#^	<0.001
% predicted R5-R20 (median, IQR)	160.4 (104.4, 279.1)	263.2 (157.8, 408.2) *	98.5 (51.5, 161.4) *^,#^	<0.001
X5 (cmH_2_O/L/s) (median, IQR)	−1.12 (−1.98, −0.58)	−1.95 (−2.68, −1.31) *	−0.98 (−1.36, −0.54) ^#^	<0.001
% predicted X5 (median, IQR)	112.6 (73.2, 178.1)	189.0 (140.9, 276.8) *	95.5 (59.9, 138.3) ^#^	<0.001
Fres (Hz)	17.1 ± 6.4	22.5 ± 5.7 *	12.8 ± 4.2 *^,#^	<0.001
% predicted Fres	122.2 ± 35.1	166.5 ± 48.7 *	100.8 ± 28.4 *^,#^	<0.001
AX (cmH_2_O/L) (median, IQR)	5.36 (2.54, 13.40)	16.10 (7.16, 25.25) *	3.96 (2.13, 5.99)	<0.001
% predicted AX (median, IQR)	144.5 (91.0, 285.1)	441.1 (213.9, 776.4) *	116.9 (61.8, 186.5)	<0.001

**Note:** Data are mean ± standard deviation (SD) unless otherwise stated; *p*-value from analysis of variance (ANOVA) or Chi-square; *, *p* < 0.017 compared with PRISm; ^#^, *p* < 0.017 compared with COPD; *p*-value of difference between group was significant with adjusted level of significance; (0.05/3 = 0.017). **Abbreviations:** PRISm, preserved ratio impaired spirometry; COPD, chronic obstructive pulmonary disease; FVC, forced vital capacity; FEV_1_, forced expiratory volume in the first second; FEF25-75%, forced expiratory flow at 25–75% of FVC; FR, fixed ratio; R5, resistance at 5 Hz; R20, resistance at 20 Hz; R5-R20, heterogeneity of resistance between R5 and R20; Fres, resonant frequency; X5, reactance at 5 Hz; AX, the area under reactance curve between 5 Hz and resonant frequency.

**Table 5:** Diagnostic Performances of IOS Parameters for Detection of PRISm using the LLN Criteria (FEV_1_/FVC ≥ LLN and %predicted of FEV_1_ < LLN) and the FR criteria (FEV_1_/FVC ≥ 0.7 and %predicted of FEV_1_ < 80%) between PRISm and Healthy Subjects.

IOS Variables	AUC	95%CI
**Detection of PRISm using the LLN criteria**		
%predicted of R5-R20	0.75	0.64, 0.85
%predicted of AX	0.66	0.51, 0.81
%predicted of Fres	0.65	0.51, 0.79
%predicted of X5	0.54	0.36, 0.73
%predicted of R5	0.54	0.37, 0.70
%predicted of R20	0.41	0.26, 0.59
**Detection of PRISm using the FR criteria**		
%predicted of R5-R20	0.72	0.63, 0.81
%predicted of AX	0.61	0.49, 0.73
%predicted of Fres	0.67	0.57, 0.77
%predicted of X5	0.61	0.49, 0.73
%predicted of R5	0.58	0.47, 0.69
%predicted of R20	0.47	0.35, 0.58

**Abbreviations:** IOS, impulse oscillometry; PRISm, preserved ratio impaired spirometry; LLN, lower limit of normal; FR; fixed ratio; AUC, area under the curve; R5, resistance at 5 Hz; R20, resistance at 20 Hz; R5-R20, heterogeneity of resistance between R5 and R20; Fres, resonant frequency; X5, reactance at 5 Hz; AX, area under reactance curve between 5 Hz and resonant frequency; CI, confidence interval.

**Table 6:** Cut-off Value of %predicted of R5-R20 for Detection of PRISm by using the LLN Criteria (FEV_1_/FVC ≥ LLN and %predicted of FEV_1_ < LLN) and the FR criteria (FEV_1_/FVC ≥ 0.7 and %predicted of FEV_1_ < 80%) between PRISm and Healthy Subjects.

%Predicted of R5-R20	Cut-Off Value	Sensitivity (95% CI)	Specificity (95% CI)	+LR (95% CI)	−LR (95% CI)	Odd Ratio (95% CI)
**Detection of PRISm using the LLN criteria**						
%predicted of R5-R20	≥120	70.6 (44.0, 89.7)	60.0 (48.4, 70.8)	1.76 (1.17, 2.65)	0.49 (0.23, 1.05)	3.6 (1.2, 10.8)
**Detection of PRISm using the FR criteria**						
%predicted of R5-R20	≥120	67.5 (50.9, 81.4)	60.0 (48.4, 70.8)	1.69 (1.20, 2.38)	0.54 (0.34, 0.88)	3.1(1.4, 6.9)

**Abbreviations:** PRISm, preserved ratio impaired spirometry; LLN, lower limit of normal; FR; fixed ratio; R5-R20, heterogeneity of resistance between R5 and R20; +LR, positive likelihood ratio; −LR, negative likelihood ratio; CI, confidence interval.

## Data Availability

The datasets used and/or analyzed during the current study are available from the corresponding author upon reasonable request.
